# Effectiveness of a Text Messaging–Based Intervention Targeting Alcohol Consumption Among University Students: Randomized Controlled Trial

**DOI:** 10.2196/mhealth.9642

**Published:** 2018-06-25

**Authors:** Kristin Thomas, Ulrika Müssener, Catharina Linderoth, Nadine Karlsson, Preben Bendtsen, Marcus Bendtsen

**Affiliations:** ^1^ Division of Community Medicine Department of Medical and Health Sciences Linköping University Linköping Sweden; ^2^ Department of Medical Specialist Linköping University Motala Sweden

**Keywords:** alcohol consumption intervention, text message-based intervention, university students, brief intervention

## Abstract

**Background:**

Excessive drinking among university students is a global challenge, leading to significant health risks. However, heavy drinking among students is widely accepted and socially normalized. Mobile phone interventions have attempted to reach students who engage in excessive drinking. A growing number of studies suggest that text message–based interventions could potentially reach many students and, if effective, such an intervention might help reduce heavy drinking in the student community.

**Objective:**

The objective of this study was to test the effectiveness of a behavior change theory–based 6-week text message intervention among university students.

**Methods:**

This study was a two-arm, randomized controlled trial with an intervention group receiving a 6-week text message intervention and a control group that was referred to treatment as usual at the local student health care center. Outcome measures were collected at baseline and at 3 months after the initial invitation to participate in the intervention. The primary outcome was total weekly alcohol consumption. Secondary outcomes were frequency of heavy episodic drinking, highest estimated blood alcohol concentration, and number of negative consequences attributable to excessive drinking.

**Results:**

A total of 896 students were randomized to either the intervention or control group. The primary outcome analysis included 92.0% of the participants in the intervention group and 90.1% of the control group. At follow-up, total weekly alcohol consumption decreased in both groups, but no significant between-group difference was seen. Data on the secondary outcomes included 49.1% of the participants in the intervention group and 41.3% of the control group. No significant between-group difference was seen for any of the secondary outcomes.

**Conclusions:**

The present study was under-powered, which could partly explain the lack of significance. However, the intervention, although theory-based, needs to be re-assessed and refined to better support the target group. Apart from establishing which content forms an effective intervention, the optimal length of an alcohol intervention targeting students also needs to be addressed in future studies.

**Trial Registration:**

International Standard Randomised Controlled Trial Number ISRCTN95054707; http://www.isrctn.com/ISRCTN95054707 (Archived by WebCite at http://www.webcitation.org/70Ax4vXhd)

## Introduction

Excessive drinking among college and university students remains a challenge despite numerous efforts to reduce students’ drinking habits [1,2]. Only a minority of students seek advice and support from student health care (SHC) services, and it is therefore urgent to find new means of reaching students who drink excessively [3].

Previous studies suggest that text messaging can be a cost-effective mode of delivery of interventions targeting health behavior change [4-6]. Text messaging–based, or text-based, interventions are effective in supporting behavior change in various areas such as weight loss, smoking cessation, and diabetes management [7]. A recent review of 36 studies on the use of text messages in mental health concluded that text messaging is a promising tool for managing excessive drinking and other mental health conditions [8].

Furthermore, text-based interventions have several advantages compared with other digital interventions (eg, web portals requiring users to log in multiple times) because they allow for high accessibility, that is messages are likely to be read within minutes of being received, receiving and reading messages requires limited time and effort by the user [9-11], and they can enable continuous, real-time, brief support in a real-world setting [12]. A common challenge in technology-based interventions is participant retention; however, in a review by Head et al [4], the retention in text-based interventions was approximately 70%. However, retention per se does not reflect engagement and adherence to an intervention and can only be seen as a proxy for both [13].

Despite the omnipresence of mobile phones with text messaging capacity, few studies have explored the potential of text messaging for changing risky drinking behaviors [4-6]. In a recent 2013 review, none of the 19 randomized controlled trials (RCTs), from 13 countries, addressed alcohol consumption [4]. Some years later, in a review of text-based interventions for young adults, 3 out of 14 studies included alcohol consumption. All 3 studies were feasibility trials with few participants [5]. A year later, a systematic review on mobile technology-based interventions for adult users of alcohol identified 8 studies, 5 of which featured interventions that were primarily delivered through text messaging [6]. However, the interventions considerably varied with regards to the length and dosage, with the longest being a 6-week intervention. Dosage varied from weekly text messages to text messages 4-6 times daily. However, most studies reported significant behavior outcomes, although outcome measures greatly varied among interventions [6]. Moreover, the few studies of text-based interventions that targeted alcohol consumption mostly lacked theory-guided content and typically included a small number of participants [4-6].

Despite the promising potential of text-based alcohol interventions, it is unclear how their effectiveness can be optimized; for example, message content and structure [14-15] or how users’ interest and adherence can be maximized [16]. User compatibility is seldom evaluated and, at best, is performed after delivery of the intervention [16]. The intervention in the present study was built upon behavior change theory using a formative development design involving users in the target group as well as experts in alcohol overuse prevention [17-18].

The aim of the present RCT was to explore the effectiveness of a theory-based intervention, using text messages, targeting college and university students.

## Methods

### Study Design

This study was a two-arm RCT. Participants were randomized to either an intervention group or a treatment as usual group (control). Outcome measures were collected at baseline and at follow-up.

### Study Setting and Inclusion Criteria

All students at 14 universities and colleges in Sweden were simultaneously invited to take part in the study. Inclusion criteria were as follows: drinking at least 4 standard drinks (females) or 5 standard drinks (males) on at least 2 occasions per month; willingness to attempt to reduce alcohol consumption; owning a mobile phone; and willingness to disclose their mobile phone number. A standard drink of alcohol in Sweden is defined as 12 g of pure alcohol. A protocol article describes the study in more detail [17].

### Follow-Up

Follow-up was carried out simultaneously for all participants 3 months after the initial invitation to the study. All participants received an email including a link to a follow-up questionnaire that investigated the primary and secondary outcomes. Two reminders via email, 1-week apart, were sent to non-responders. In addition, participants who continued to not respond received a text message every second day for 6 days (ie, 3 additional reminders). These text messages only included a single question investigating the primary outcome (total weekly consumption). Finally, non-responders were contacted via telephone (maximum of 10 calls). Again, only the primary outcome was investigated.

### Intervention

The intervention was a 6-week automated text message–based program with a total of 62 messages, as described in more detail in previous papers [17,18]. The intervention was developed using formative methods including focus groups with students, an expert panel with students and professionals, and a behavior change technique analysis [18]. Twenty-three behavior change techniques were identified in the final version of the intervention, using the taxonomy developed by Michie et al [19]. Some techniques were used in more than 1 message or across 2 messages. The techniques aimed to motivate students to reduce their alcohol consumption, address self-regulation, increase self-efficacy, and increase students’ awareness of social and professional support.

The first 4 weeks of the program had a higher frequency of messages, 9 in each week, followed by 7 messages in weeks 2-5, and 5 messages in week 6. Two messages were repeated at the start of each week; students were asked to report via a text message the number of drinks they had consumed the previous week. Subsequently, they received a second text message including feedback on their performance in relation to their goal set at the start of the intervention. These paired messages were repeated every Sunday [17].

### Control

The control group was offered treatment as usual. At present, the typical practice at the SHCs, besides motivating advice delivered face-to-face, is to recommend a website to the students where they can estimate their alcohol consumption, receive feedback on their drinking levels and more information on health consequences of drinking. Participants allocated to the control group were informed of this and told that they would gain access to the intervention once the main trial had ended. No additional prompts or reminders about the websites were given [17].

### Outcome Measures

A baseline questionnaire included 9 items investigating (1) age (continuous), (2) gender (female/male), (3) relationship status (single/in a relationship). Four items investigated outcome measures (4) total weekly alcohol consumption during a typical week computed as the sum of alcohol consumption (in standard units) for each day in a typical week, (5) number of days with heavy episodic drinking (HED) during the most recent month, (6) estimated blood alcohol concentration (eBAC) during the heaviest drinking occasion the most recent month, and (7) number of negative consequences caused by drinking alcohol during the most recent month. In addition, students were asked to (8) state a goal for reducing their weekly alcohol consumption. Finally, students were asked to (9) specify the mobile phone number to which they wished to receive the intervention [17].

We estimated eBAC based on reported greatest alcohol intake during a single-drinking occasion the past month. The time spent consuming the alcohol, weight, and gender were also evaluated. A variation of the Widmark formula developed for road safety research was used to compute eBAC [20].

The follow-up questionnaire included 4 questions investigating the primary and secondary outcomes: (1) total weekly alcohol consumption during a typical week, (2) HED during the last month, (3) eBAC during the last month, and (4) number of negative consequences caused by drinking alcohol during the last month [17]. Participants responding to the email follow-up were asked to estimate their weekly consumption every day, and researchers summed these estimates to create the primary outcome variable. Participants responding via text or telephone were asked to estimate their consumption over a week. No recall methods were used at baseline or follow-up.

### Recruitment Process

Students were invited to participate through an email from their local SHC including 2 reminders issued at one and 2 weeks after the initial invitation. Students were allowed to respond up to 7 days after the final reminder. No other advertisement strategy was used. The invitation email aimed to attract and reach students who thought they drank too much and were willing to reduce their consumption. Students could choose between 2 links in the email: (1) “Yes, I would like to know more about the study” or (2) “No, I do not wish to take part in the study or receive any reminders.” Students who clicked on the first link were transferred to an eligibility criteria screen.

Students who did not meet the inclusion criteria were automatically referred to a screen including tips from a website where they could find support if needed. Students who met the inclusion criteria were automatically referred to an informed consent screen that also included detailed information on the study and participation. Interested students gave their informed consent to participate by clicking on a link that automatically transferred them to the baseline questionnaire.

**Figure 1:**
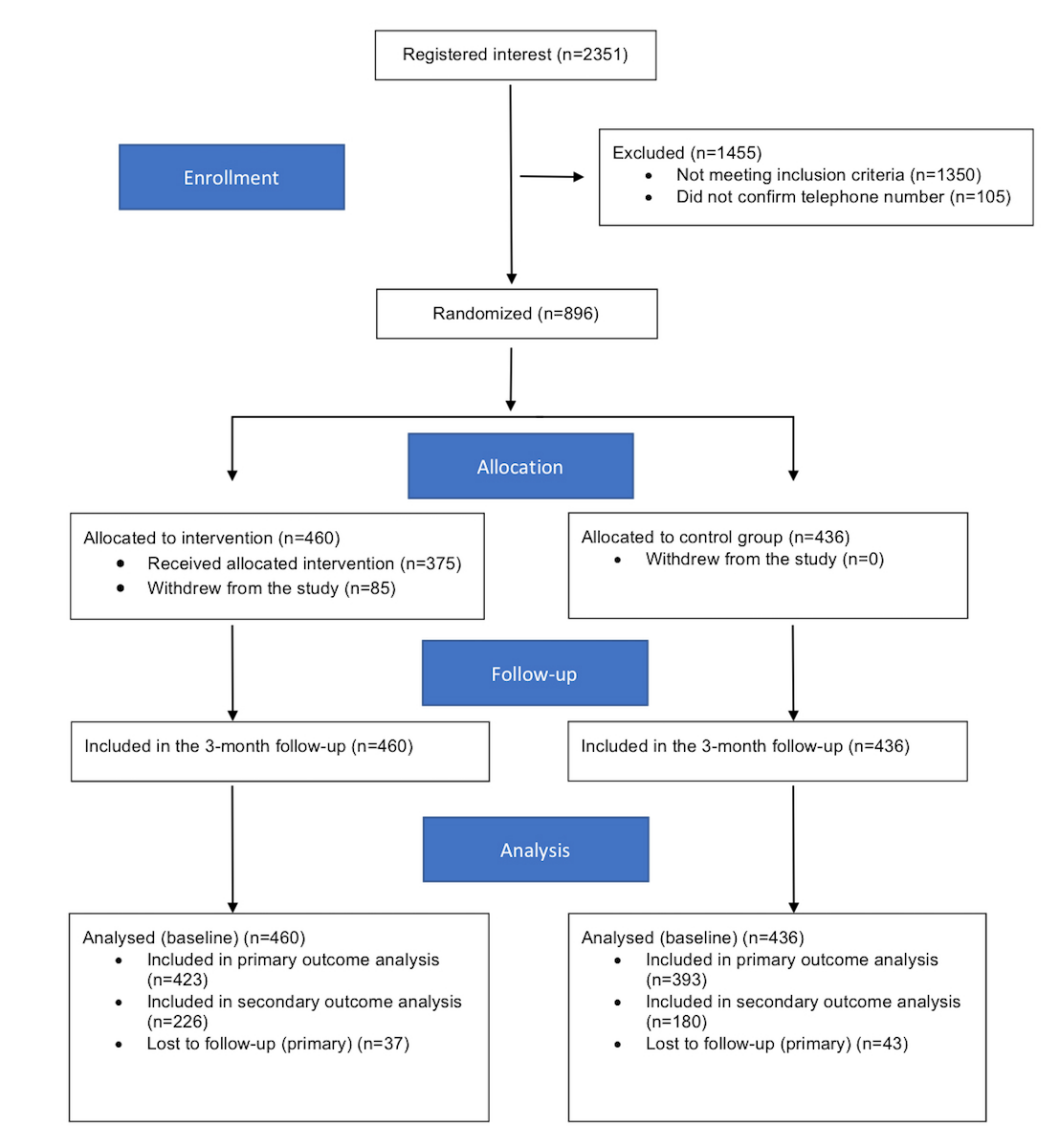
The Consolidated Standards of Reporting Trials (CONSORT) 2010 flow diagram.

After completion of the baseline questionnaire, students were asked to provide their mobile phone number. Students then immediately received a text message asking them to confirm their mobile phone number by responding “start.” All students who confirmed their mobile phone number were randomized to either the intervention or control group. A text message was sent to each participant with information about which group they were allocated to. Figure 1 depicts a flowchart of the recruitment procedure of the study.

### Randomization and Blinding

Each participant was allocated either number 1 or 2 with equal probability using Java’s built-in random number generator (java.util.Random). Randomization was thus fully computerized, did not use any strata or blocks, and was not possible to subvert, because this and all subsequent study processes were fully automated.

### Power Calculation

To detect a standardized effect size of 0.15 between the 2 groups at 3-months’ follow-up with a 5% significance level and 80% power, we calculated that we would require 699 individuals analyzed per group, ie, a total of 1398 individuals. Assuming a 3-month follow-up rate of 80%, we needed 874 per group (ie, a total of 1748 individuals).

### Statistical Analysis

All statistical analyses were performed as described in the protocol article [17]. The data was examined graphically for outliers but no such outliers were found.

Baseline characteristics of responders were compared between randomized groups using the chi-squared test or Fisher’s exact test for comparison of proportion, and Student’s t test for comparison of means.

All outcome analyses were compared between the 2 randomized groups (both with the same follow-up time) under the intention-to-treat principle (that is, all randomized individuals were included in their originally randomized groups). Total weekly consumption, eBAC, and the number of negative consequences were skewed as seenby visual inspection of histograms or Q-Q plots. Due to the fact that they these outcomes were skewed, eBAC was log-transformed and analyzed with linear regression, and weekly alcohol consumption and negative consequences were analyzed with negative binomial regression. Group specific means were reported for log-transformed variables asgeometric means with back-transformed standard deviations, and for variables analyzed by negative binomial regression as back-transformed means and standard deviations. Frequency of HED occasions was analyzed by ordered logistic regression. All regression analyses were first performed unadjusted, and then adjusted for weekly alcohol consumption at baseline, age (analyzed as continuous variable), university

Missing outcome data were initially handled by a complete-cases analysis, which assumes that the data are missing at random (MAR). In a sensitivity analysis, we explored the plausibility of the MAR assumption by regressing the primary outcome (weekly alcohol consumption) on the number of follow-up attempts needed before an individual responded. Any significant association could be evidence against the MAR assumption.

Attrition was investigated for statistically significant differences between the study groups regarding completion of the follow-up questionnaire. This was done by comparing baseline characteristics among participants who did and did not respond at follow-up. Any significant associations could provide possible evidence against the MAR assumption.

None of the primary analyses were completed with imputed values. In a sensitivity analysis we carried over baseline values and used follow-up group means for the primary outcomes. No sensitivity analysis or imputation was done for secondary outcomes. Effect modification tests for total weekly consumption as baseline, age, university, and gender were undertaken for the primary outcome only. All analyses were performed as two-sided tests with a 5% level of significance.

## Results

### Overview

A total of 896 participants were randomized: 460 (51.3%) to the intervention group and 436 (48.7%) to the control group. The number of female participants was slightly more in both groups. A summary of all baseline characteristics is given in Table 1. University code represents an id for the university the participant attends, and eBAC is reported *per mille*. There were no significant differences in any of the sociodemographic characteristics or drinking variables.

### Primary Outcome Analysis

The primary outcome analysis was done on a total of 423 (92.0%) randomized participants in the intervention group and 393 (90.1%) in the control group (*P*=.34 by chi-squared test). Weekly alcohol consumption decreased in both groups with no statistically significant difference between groups (Table 2). There was no evidence of a statistically significant effect modifier between baseline variables (weekly alcohol consumption at baseline, age, university, and gender) and treatment group at the 5% level of significance.

### Secondary Outcome Analysis

Secondary outcome data were available only for participants completing the follow-up by email and included 226 (49.1%) of the participants in the intervention group and 180 (41.3%) in the control group (*P*=.02 by chi-squared test). Both groups exhibited decreased frequency of HED with no statistically significant differences between the groups (Table 2). The eBAC declined between baseline and follow for both groups, from around 1.4 to 0.9, with no statistically significant difference between groups. The number of negative consequences associated with excessive drinking declined from just above 3 to just above 2, with no statistically significant differences between groups.

### Sensitivity Analysis

**Figure 2:**
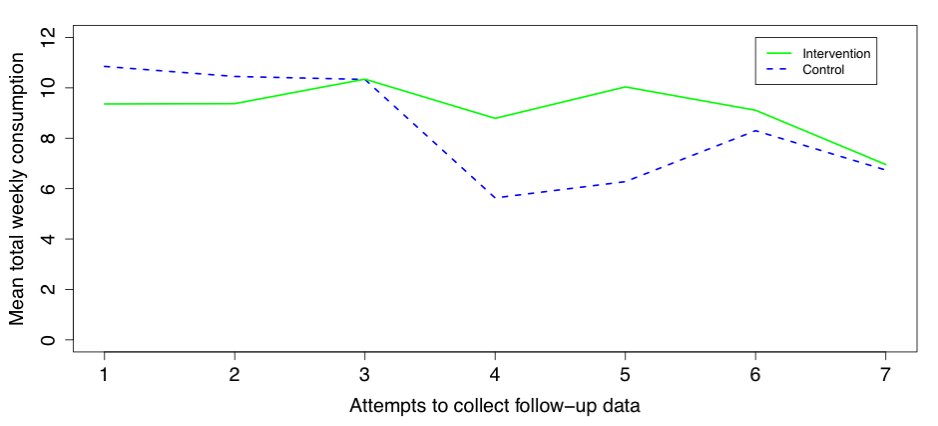
Mean total weekly consumption reported at the different attempts to collect follow-up data.

Based on negative binomial regression, there was a statistically significant decrease in weekly alcohol consumption at follow-up as a function of number of attempts before answering the follow-up (incidence rate ratio [IRR] 0.94 (0.92,0.96), *P*<.001). This trend can be seen when plotting the mean total weekly consumption reported at the respective attempts, which is depicted in Figure 2. No changes to the primary analyses, with respect to statistical significance, were found when the analyses were redone under the assumption that missing data were equal to baseline values (carry over) nor when setting missing values to respective group follow-up means.

Attrition was investigated for statistically significant differences between the study groups regarding completion of the follow-up questionnaire. Baseline characteristics were compared between participants who did and did not respond at follow-up. No statistically significant differences were found among those for which primary outcome data was collected. However, there were statistically significant differences for secondary outcomes: non-responders were younger (*P*<.001), more often single (*P*=.03), and had a higher eBAC (*P*=.02) than responders

### Post-Hoc Analysis

Because there was a statistical association between number of attempts to record follow-up data and the mean weekly consumption at these follow-up attempts we re-conducted the primary analysis using data collected only via the email (ie, attempts 1, 2, and 3), leaving aside follow-up data collected through text messages and telephone. In this analysis, a much lower *P*-value was recorded (IRR 0.90 (0.80, 1.02), *P*=.11), although not below the predefined 0.05 threshold. No such reductions of *P*-values were found when only considering text message follow-ups or telephone calls.

**Table 1:** Comparison of groups at baseline.

Variable			Intervention (n=460)	Control (n=436)	*P* value
Gender (females), n (%)			265 (57.6)	244 (56.0)	.62
Age (years), mean (SD)^a^			25.3 (6.7)	25.6 (6.8)	.43
**Age (years), categorical, n (%)**					.36
	<21		119 (26.0)	110 (25.4)	
	21-25		207 (45.2)	175 (40.4)	
	26-30		76 (16.6)	85 (19.6)	
	>31		56 (12.2)	63 (14.5)	
Marital status (single), n (%)			288 (62.9)	256 (59.1)	.25
**University code, n (%)**					.89
	1		55 (12.0)	45 (10.3)	
	2		13 (2.8)	9 (2.1)	
	3		30 (6.5)	36 (8.3)	
	4		47 (10.2)	47 (10.8)	
	5		27 (5.9)	30 (6.9)	
	6		41 (8.9)	42 (9.6)	
	7		10 (2.2)	12 (2.8)	
	8		12 (2.6)	12 (2.8)	
	9		18 (3.9)	12 (2.8)	
	10		32 (7.0)	31 (7.1)	
	11		5 (1.1)	10 (2.3)	
	12		157 (34.1)	141 (32.3)	
	13		13 (2.8)	9 (2.1)	
**Alcohol parameters**					
	Weekly alcohol consumption, mean (SD)		13.90 (8.43)	13.66 (8.30)	.67
	**Frequency of HED^b^, n (%)**				
		2-3 times a month	131 (28.5)	130 (29.8)	.77
		Approximately 1 time a week	241 (52.4)	218 (50.0)	
		More than 1 time a week	88 (19.1)	88 (20.2)	
	Highest eBAC^c^, mean (SD)		1.32 (0.86)	1.38 (0.94)	.32
	Number of negative consequences of excessive drinking^d^, mean (SD)		3.19 (1.96)	3.17 (1.95)	.88

^a^Intervention (n=458); control (n=433).

^b^HED: heavy episodic drinking (how often, during the past 3 months, have you consumed 4 (for females) / 5 (for males) standard drinks on one occasion?).

^c^eBAC: estimated blood alcohol concentration.

^d^Includes negative consequences on studies, academic results, finances, social relationships, gender, regrettable situations, mental health, injuries, conflict, violence, and sleep.

**Table 2:** Drinking outcomes at follow-up and analysis of treatment effect intervention versus control.

Outcome		Intervention (n=423)		Control (n=393)		Unadjusted ratio (95% CI)		*P* value		Adjusted^a^ ratio (95% CI)		*P* value	
**Primary outcomes, mean (SD)**													
	Weekly alcohol consumption		8.75 (7.28)		8.55 (7.13)		1.02 (0.91, 1.15)^c^		.70		0.99 (0.90, 1.09)^c^		.83
**Secondary outcomes^d^, mean (SD)**													
	Number of negative consequences of excessive drinking		2.17 (1.56)		2.33 (1.63)		0.93 (0.81, 1.07)^c^		.33		0.92 (0.81, 1.06)^c^		.24
**Frequency of HED^e^, n (%)**		—		—		0.92 (0.64, 1.31)^f^		.65		0.85 (0.58, 1.22)^f^		.37	
	Never		11 (4.9)		7 (3.9)		—		—		—		—
	Less than once a month		9 (4.0)		8 (4.4)		—		—		—		—
	Approximately once a month		41 (18.1)		30 (16.7)		—		—		—		—
	2-3 times per month		57 (25.2)		46 (25.6)		—		—		—		—
	Approximately once a week		91 (40.3)		74 (41.1)		—		—		—		—
	More than once a week		17 (7.5)		15 (8.3)		—		—		—		—
Highest eBAC^g^, mean (SD)		0.96 (0.80)		0.93 (0.80)		1.02 (0.94, 1.10)^h^		.66		0.99 (0.92, 1.07)^h^		.85	

^a^Adjusted for weekly alcohol consumption at baseline, age, university, and gender. Includes negative consequences on studies, academic results, finances, social relationships, gender, regrettable situations, mental health, injuries, conflict, violence, sleep.

^b^Values refer to intervention compared with control.

^c^Ratio of means, by negative binomial regression.

^d^Intervention (n=226); control (n=180).

^e^HED: heavy episodic drinking.

^f^Odds ratio, by ordered logistic regression.

^g^eBAC: estimated blood alcohol concentration.

^h^Ratio of geometric means, by linear regression after log transformation.

## Discussion

We could not demonstrate any statistically significant between-group differences relative to the novel intervention or treatment as usual. This is one of the largest studies on the subject of alcohol consumption reduction performed so far [6]; however, we did not reach a sufficient number of participants according to initial power calculations. This could partially explain the lack of statistical difference between the groups. Regulations at the participating universities and colleges required us to limit email reminders; therefore, it was not possible for us to continue to email and recruit more students. Apart from this, the intervention itself needs to be re-assessed and refined to better support the target group for reducing alcohol consumption.

The primary analyses were performed under the assumption of MAR. The attempts model and differences between responders and non-responders, with respect to the secondary outcomes, question this MAR assumption. Although no changes in the primary analyses were found under different assumptions about the missing data (carry over and group means), these trends are still interesting to discuss. There could potentially be a bias that late responders drink less, ie, participants were not willing to engage because they did not think that their alcohol consumption was a problem. There could also be a time component where general alcohol consumption in the study population decreased (the follow-ups conducted over telephone happened several weeks after the first follow-up email). The high response rate alleviates the problem of these 2 cases to some degree because if the trend continues beyond 7 attempts, only a few cases would report even lower consumption rates. Given that the trend was present in both groups, this bias was alleviated in group comparisons. However, one might question if the aim of measuring the 3-month effect of the intervention is still relevant in light of this trend. The post-hoc analysis using only data collected via email (the first 3 attempts) lowers the *P*-value for a positive effect of the intervention, suggestive of a 3-month positive effect. However, one must be aware that the *P*-value was not less than the predefined level, and attrition analysis showed that there were significant differences between responders and non-responders when only considering follow-up data collected via email.

There are 2 more potential reasons why the attempt model showed a statistically significant trend. First, we asked email responders to estimate every day how many standard drinks they consumed, and then we summed these estimates to get the total weekly consumption. However, for practical reasons, we asked text and phone responders to directly estimate their total weekly consumption, and such differences in reporting may introduce bias to estimates given by responders. Second, the mode by which the data was collected may also bias the responses, especially when calling respondents because they may feel social pressure to report more positive answers than the truth. If either of these are the reasons for the trend, then the results in Table 2 are highly suspicious.

In the invitation to the study, we emphasized that participants should be willing to try to reduce their alcohol consumption. This was stated as information about the study, and in the informed consent text. There was not an explicit question about willingness in the baseline questionnaire. However, participants were asked to state a presumptive reduction goal in the baseline questionnaire. After this, the randomization took place. This means that all participants in the control group stated to what extent they wished to reduce their consumption. This may have influenced the outcome in the control group; however, the study aimed to investigate whether support via text messages helped reduced alcohol consumption, compared with routine practice.

The study has several limitations. This study was under-powered, and the MAR assumption was questionable. Among early responders (all using the same mode of response, estimating weekly consumption every day), the *P*-value for a positive effect of the intervention decreased but not enough to be statistically significant. The MAR assumption should still be questioned in this subset of the data. Another limitation is the proportion of participants exposed to the whole intervention because approximately 18% dropped out before the intervention finished. This might also have influenced the negative findings in the study.

A major strength of the study was the formative development design that entailed revision of all messages based on both user and expert feedback. Another strength of the study was the use of BCT analysis that elucidated the theory base of the messages, giving readers a better understanding of what the intervention entailed and enabling comparison with other interventions [21]. However, despite using 23 behavior change techniques that have been identified as effective for mobile interventions targeting alcohol interventions [20], we observed no treatment effect.

The inclusion of a weekly normative feedback was also hypothesized to be effective based upon a Cochrane review that showed receiving normative feedback had a small effect on reducing alcohol consumption in student populations [22]. Furthermore, self-monitoring is associated with improved effectiveness of brief interventions [19]. An earlier study also found that reporting alcohol use on a daily basis reduced drinking among heavy drinkers by 20% [23]. We could however not show any effect despite including the above elements in the intervention.

In most previous studies, the intervention had a longer duration (ie, around 12 weeks). This is twice as long as the 6 weeks used in the current study [6]. We still do not know whether a 6-week intervention is too short for supporting behavior change.

The present study did not demonstrate any differences in effects on alcohol consumption between a 6-week theory-based intervention and treatment as usual, among college and university students. Future studies should consider the length of the intervention and also whether techniques used in face-to-face interventions could be applied in text-based interventions [21].

## Appendix

Multimedia Appendix 1 CONSORT‐EHEALTH checklist (V 1.6.1).

## Abbreviations

eBAC: estimated blood alcohol concentration
HED: heavy episodic drinking
IRR: incidence rate ratio
MAR: missing at random
RCT: randomized controlled trial
SHC: student health care
